# C-Reactive Protein: The Quintessential Marker of Systemic Inflammation in Coronary Artery Disease—Advancing toward Precision Medicine

**DOI:** 10.3390/biomedicines11092444

**Published:** 2023-09-02

**Authors:** Emanuel Amezcua-Castillo, Héctor González-Pacheco, Arturo Sáenz-San Martín, Pablo Méndez-Ocampo, Iván Gutierrez-Moctezuma, Felipe Massó, Daniel Sierra-Lara, Rashidi Springall, Emma Rodríguez, Alexandra Arias-Mendoza, Luis M. Amezcua-Guerra

**Affiliations:** 1 Escuela Nacional Preparatoria No. 6 Antonio Caso, Universidad Nacional Autónoma de México, Mexico City 04100, Mexico; 323059378@alumno.enp.unam.mx; 2 Coronary Care Unit, Instituto Nacional de Cardiología Ignacio Chávez, Mexico City 14080, Mexico; hectorglezp@hotmail.com (H.G.-P.); danielsierralaram@gmail.com (D.S.-L.); aariasm@yahoo.com (A.A.-M.); 3 School of Medicine, Universidad Autónoma Metropolitana–Xochimilco, Mexico City 14387, Mexico; saenzsma@gmail.com (A.S.-S.M.); xpamo18x@gmail.com (P.M.-O.); ivan.moctezuma313@gmail.com (I.G.-M.); 4 Translational Research Unit, Instituto Nacional de Cardiología Ignacio Chávez, Mexico City 14080, Mexico; f_masso@yahoo.com (F.M.); emarod2@yahoo.com.mx (E.R.); 5 Department of Immunology, Instituto Nacional de Cardiología Ignacio Chávez, Mexico City 14080, Mexico; raspringall@yahoo.com; 6 Health Care Department, Universidad Autónoma Metropolitana–Xochimilco, Mexico City 14387, Mexico

**Keywords:** C-reactive protein, coronary artery disease, atherosclerotic cardiovascular disease, inflammation, precision medicine, personalized cardiology

## Abstract

Atherosclerotic cardiovascular disease (CVD) remains the leading cause of mortality worldwide. While conventional risk factors have been studied and managed, CVD continues to pose a global threat. Risk scoring systems based on these factors have been developed to predict acute coronary syndromes and guide therapeutic interventions. However, traditional risk algorithms may not fully capture the complexities of individual patients. Recent research highlights the role of inflammation, particularly chronic low-grade inflammation, in the pathogenesis of coronary artery disease (CAD). C-reactive protein (CRP) is an inflammatory molecule that has demonstrated value as a predictive marker for cardiovascular risk assessment, both independently and in conjunction with other parameters. It has been incorporated into risk assessment algorithms, enhancing risk prediction and guiding therapeutic decisions. Pharmacological interventions with anti-inflammatory properties, such as statins, glucagon-like peptide-1 agonists, and interleukin-1 inhibitors, have shown promising effects in reducing both cardiovascular risks and CRP levels. This manuscript provides a comprehensive review of CRP as a marker of systemic inflammation in CAD. By exploring the current knowledge surrounding CRP and its implications for risk prediction and therapeutic interventions, this review contributes to the advancement of personalized cardiology and the optimization of patient care.

## 1. Introduction

Atherosclerotic cardiovascular disease (CVD) remains the leading cause of mortality worldwide. While significant progress has been made in understanding and managing conventional risk factors such as hypertension, diabetes, dyslipidemia, and smoking, CVD continues to pose a significant global health challenge [1]. Risk scoring systems incorporating these conventional risk factors have been developed to predict acute coronary syndromes (ACS) and guide therapeutic interventions. Moreover, dietary and pharmacological interventions targeting cardiovascular risk factors have demonstrated substantial benefits in the prevention and progression of coronary artery disease (CAD) [2]. In addition, public policies addressing tobacco use have contributed to reducing the incidence of ACS and the progression of atherosclerotic CVD [3]. However, it is increasingly recognized that traditional risk algorithms may not fully capture the complexities of individual patients. In recent years, inflammation has emerged as a crucial component in the pathogenesis of CVD, with numerous studies elucidating the intricate relationship between inflammation and CAD [4].

Chronic low-grade inflammation, in particular, has been acknowledged as a contributing factor in the initiation, progression, and complications of various cardiovascular conditions, including atherosclerosis, ACS, and heart failure. Interestingly, a reciprocal causality has been unveiled wherein cardiovascular risk factors exhibit the ability to incite inflammatory responses. Indeed, hypertension, dyslipidemia, smoking, diabetes, and obesity have been attributed to the ability to trigger and sustain inflammatory responses. The secretion of proinflammatory molecules may trigger a cascade of events that culminate in the destabilization of atherosclerotic lesions. This reciprocal interplay between inflammation and the pathogenesis of CAD elucidates a compelling rationale for the comprehensive management of cardiovascular risk factors with interventions that improve cardiometabolic status and decrease hypercoagulability while exerting anti-inflammatory effects [4,5].

To effectively combat CVD, it is imperative to understand the molecular and cellular processes driving the inflammatory response. This understanding is paving the way for the development of targeted anti-inflammatory interventions and bridging the gap between basic research and clinical cardiology. The shift toward translational cardiology enables the transfer of knowledge from the laboratory bench to the patient’s bedside, with the ultimate goal of achieving precision cardiology. The realization of personalized cardiology, wherein interventions are tailored to individual patients based on their unique clinical, thrombotic, and inflammatory profiles, holds great promise. By incorporating insights from the inflammatory milieu underlying each patient, we can enhance risk prediction and refine therapeutic strategies and patient outcomes [6].

This manuscript aims to provide a comprehensive review of the role of C-reactive protein (CRP) in a unique molecule functioning as a quintessential marker of systemic inflammation in the era of precision medicine. By examining the current knowledge surrounding CRP and its implications for risk prediction and therapeutic interventions, this review aims to contribute to the advancement of personalized cardiology and the optimization of patient care.

## 2. Inflammation as a Novel Pathogenic Paradigm in Myocardial Infarction

Acute myocardial infarction (MI) triage relies on the electrocardiographic assessment of ST-segment changes, a venerable technology in use for more than a century. In the same way, confirmation of myocardial necrosis is achieved through the measurement of molecules that result from the lysis of cardiomyocytes, such as creatine kinase (CK) and cardiac troponins. Although this strategy has shown to be useful when developing evidence-based medicine, the advent of precision medicine has brought with it the need to integrate data at the individual level, encompassing genomics, novel biomarkers, lifestyle factors, and environmental influences [7,8].

Early postmortem studies proposed that plaque rupture was the main cause of fatal MI. This led to the concept of vulnerable plaques, characterized by a large central lipid core, inflammatory cell abundance, and a thin fibrous cap. It was believed that the weakening of the collagen structure in thin-capped fibroatheroma, caused by inflammatory mechanisms, resulted in coronary atheromata instability. Consequently, efforts were made to develop methods for detecting vulnerable plaques. However, identifying high-risk plaques was proved challenging due to their low predictive value [7]. Further studies revealed that plaque rupture commonly occurs in individuals without symptomatic ACS, and only a small percentage of plaques with thin-capped fibroatheroma features actually cause ACS over an extended follow-up period [5,8]. This suggests that most thin-capped fibroatheroma plaques are clinically stable, challenging the notion of a vulnerable plaque.

**Figure 1:**
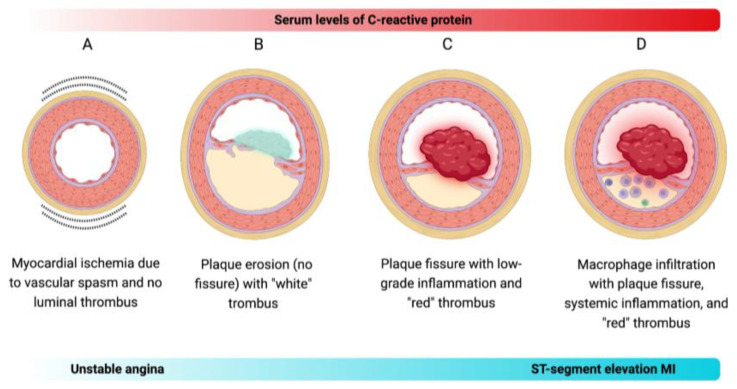
Proposed mechanistic models of ACS. (**A**) Vasospasm-induced ACS: Vasospasm, known to occur in epicardial arteries, can also impact coronary microcirculation, triggering ACS, such as unstable angina, independently of inflammation. (**B**) Plaque erosion-induced ACS: Plaque erosion is increasingly recognized as a contributor to ACS, particularly the non-ST-segment elevation MI. The thrombi formed over eroded intimal patches exhibit characteristics of platelet-rich structures, termed the “white” thrombus. (**C**) Non-inflammatory plaque rupture-induced ACS: Some atheromata may experience plaque rupture without exhibiting extensive intimal macrophage collections or elevated circulating CRP levels. This type of plaque rupture leads to the formation of fibrin-rich “red” thrombi. (**D**) Inflammatory plaque rupture-induced ACS: Plaque rupture has conventionally been considered the primary cause of ACS, especially the ST-segment elevation MI. It often occurs in plaques featuring local inflammation marked by macrophage infiltration and systemic inflammation indicated by elevated CRP levels in the blood.

Although inflammation is a major contributor to atherosclerosis development, it may not be the sole driver of the transition from stable atherosclerosis to acute thrombosis. Evidence shows that approximately half of ACS may occur even when CRP levels are normal [9]. Moreover, up to one-third of ACS cases are caused by plaque erosion, and about one-fifth of ACS events occur without detectable coronary thrombosis, indicating that functional alterations beyond thrombus formation can contribute to the pathogenesis of CAD [10]. Based on the notion that inflammation alone may not account for all transitions to MI, Crea and Libby have contextualized a new pathogenic paradigm on the various mechanisms that may cause ACS [5]. Briefly, there are four distinct mechanisms that can lead to ACS (Figure 1):

A—Vasospasm can trigger ACS in the absence of inflammation. It has long been acknowledged as a phenomenon occurring in epicardial arteries, but it can also affect coronary microcirculation.

B—Plaque erosion is becoming increasingly recognized as a cause of ACS, often resulting in non-ST-segment elevation MI. The thrombi formed over the patches of intimal erosion typically display characteristics of platelet-rich structures, namely the “white” thrombus.

C—Plaque rupture can occur in atheromata that do not exhibit extensive collections of intimal macrophages and do not show elevated levels of circulating CRP. Plaque rupture in these cases typically leads to the formation of fibrin-rich “red” thrombi.

D—Plaque rupture has traditionally been considered the primary cause of ACS, particularly the ST-segment elevation MI. It often occurs in plaques with local inflammation featured by macrophage infiltration as well as systemic inflammation indicated by elevated levels of CRP in the blood.

In the context of the inflammation-based paradigm, the assessment of ACS staging can be accomplished by evaluating CRP levels in the bloodstream. CRP is an easily accessible laboratory biomarker with a well-established standardization for measurement, and its association with CVD carries significant pathological implications. Additionally, CRP has emerged as a promising therapeutic strategy for both primary and secondary prophylaxis of CAD.

## 3. CRP Holds Potential as a Quintessential Marker of Inflammation

**Figure 2:**
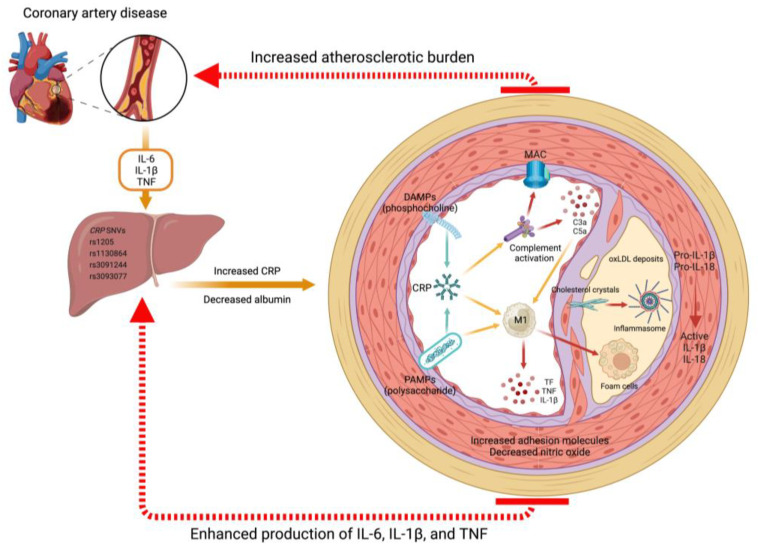
Mechanisms of CRP in atherosclerotic CVD. CRP, predominantly synthesized in the liver under the influence of proinflammatory cytokines such as IL-6, IL-1β, and TNF, plays a pivotal role in the progression of atherosclerotic CVD. Various single nucleotide variants (SNVs) can modulate CRP production. Once released into circulation, CRP exists in a pentameric form and exhibits recognition and binding capabilities to danger-associated molecular patterns (DAMPs), such as the phosphocholine found in apoptotic debris, as well as pathogen-associated molecular patterns (PAMPs), such as the lipopolysaccharide in the membrane of gram-negative bacteria. Upon activation, CRP undergoes a conformational change into functionally active monomers, leading to the initiation of the complement cascade and activation of inflammatory macrophages (M1 phenotype). In the context of chronic inflammation, these stimuli become detrimental and facilitate the deposition of oxidized low-density lipoproteins (oxLDL) and lipid-containing foam cells within the vascular sub-endothelium. The formation of cholesterol crystals in atheromatous plaques further enhances the activation of inflammasomes, which are PAMP and DAMP recognition receptors. Consequently, this process leads to increased production of IL-1β and IL-18. This inflammatory microenvironment triggers heightened production of chemoattractants and adhesion molecules while concomitantly reducing the production of nitric oxide and other vasodilatory factors. Such disruption of vascular endothelial homeostasis results in an augmented atherosclerotic burden and the development of CAD.

Produced primarily by hepatocytes and endothelial cells under the influence of interleukin (IL)-6, IL-1β, and tumor necrosis factor (TNF), CRP is a pentameric protein that belongs to the pentraxin superfamily. Its name originates from its ability to be bound to pneumococcal somatic C polysaccharide. CRP exhibits dual roles in inflammation, functioning as both pro-inflammatory and anti-inflammatory molecules [11,12]. CRP promotes the recognition and elimination of pathogens, as well as the clearance of necrotic and apoptotic debris. In addition to its high affinity for phosphocholine in the membrane of apoptotic debris, CRP activates the complement system through a pathway parallel to that of lectins, acting as an opsonin for phagocytosis (Figure 2) [13,14]. Upon activation, CRP undergoes conformational changes, adopting a monomeric form that exposes binding sites for Fc receptors and the complement system and facilitating the elimination of pathogens and damaged cells. This includes the upregulation of proinflammatory cytokines, as well as pivotal components of the inflammasome. Intriguingly, CRP may stimulate the assembly of the NLRP3 inflammasome through Fcγ receptor-mediated generation of reactive oxygen species [15,16]. Simultaneously, it is becoming evident that CRP induces endothelial activation and dysfunction by altering endothelial vasoreactivity, through a decrease in endothelial nitric oxide synthase (eNOS) activity. These effects, mediated by eNOS uncoupling, promote the release of circulating endothelial cells and endothelial microparticles, which ultimately appears to increase the risk of atherothrombosis, hypertension, and CVD [17]. Hence, CRP plays a key role in local host defense against pathogens and the efficient removal of apoptotic or damaged cells, although uncontrolled activation comes with the potential for adverse effects.

Elevated CRP levels are closely associated with both acute and chronic inflammatory conditions, spanning across a broad spectrum of disorders including infection, trauma, stroke, tissular necrosis, and neoplasms [18]. The conventional measurement techniques for CRP, commonly utilized to evaluate acute or severe inflammatory responses, often lack the requisite sensitivity to detect the subtle inflammatory nuances characterizing chronic conditions, including CVD. Conversely, the implementation of highly sensitive measurement methodologies enables the detection of high-sensitivity CRP, a refined iteration of the CRP assay. In essence, the principal distinction between CRP and high-sensitivity CRP resides in their inherent sensitivity and the expanse of clinical scenarios wherein they find application. In clinical practice, several techniques are used to measure CRP levels. The choice of a particular technique depends on factors such as the level of sensitivity required, the available resources, and the specific goals of the measurement. Immunonephelometry operates on the formation of antigen-antibody complexes and quantifies the intensity of scattered light when a laser passes through the sample. Enzyme-linked immunosorbent assay is a versatile technique that involves the use of antibodies that are bound to CRP, followed by a colorimetric reaction that quantifies the amount of CRP present. Color-flow cytometry employs fluorescently labeled antibodies to detect and quantify CRP levels within the analyzed cells or serum/plasma samples. Latex agglutination involves mixing CRP-specific antibodies with a latex suspension; in the presence of CRP in the sample, the latex particles agglutinate, allowing for visual detection or assessment through turbidity measurements. Point-of-care tests represent rapid diagnostic tools designed for immediate on-site CRP measurement. Often used in emergency departments, these tests typically use lateral flow technology to provide quick results. It is noteworthy that the information contained in this review pertains primarily to high-sensitivity CRP measurements.

CRP concentrations can be expressed in either milligrams per deciliter (mg/dL) or milligrams per liter (mg/L) [19]. Normal CRP values have been established based on reference populations, wherein 70% to 90% of individuals exhibit CRP concentrations below 3 mg/L. However, some individuals with no evident pathology may display levels up to 10 mg/L [20]. CRP levels can be interpreted as follows:

- Less than 10 mg/L: Normal or mild elevation. Minor elevations are frequently observed in conditions such as obesity, diabetes, common flu, periodontitis, and smoking. Additionally, mild CRP elevations are often linked to an increased cardiovascular risk and the development of clinical conditions, including MI, stroke, peripheral artery disease, and sudden cardiac death.
  Based on CRP levels, individuals can be classified into three CVD risk groups: CRP levels below 1 mg/L are considered low risk, CRP levels between 1 and 3 mg/L are considered moderate risk, and CRP levels exceeding 3 mg/L are considered high risk [21].
- Between 10 and 100 mg/L: Moderate elevations are observed in infectious processes, acute pancreatitis, and most autoimmune and musculoskeletal rheumatic diseases (except for systemic lupus erythematosus and systemic sclerosis) [11]. Moderate elevations are also found in ACS. In a cohort of patients with MI, González-Pacheco and colleagues reported a median CRP concentration of 12.6 mg/L (interquartile range [IQR], 5.3–37.7 mg/L) [22].
- More than 100 mg/L: Elevated CRP concentrations are observed in severe infections, often of bacterial origin, as well as in cases of severe trauma, extensive burns, and systemic vasculitis [23]. Special mention should be made for SARS-CoV-2 infection. For patients with severe COVID-19, median CRP concentrations of 149 mg/L (IQR, 71–257 mg/L) have been reported [24].

## 4. Ethnic and Gender Differences in CRP Levels

Cardiovascular risk assessment tools are primarily based on studies consisting predominantly of Caucasian men, which may generate inaccuracies when calculating the cardiovascular risk for populations with different genetics or ethnic-racial backgrounds, or for women.

Khera and colleagues investigated the differences in CRP levels based on ethnic-racial background and gender. This study involved 2749 participants, categorized into four groups: Afro-American women, Afro-American men, Caucasian women, and Caucasian men. The findings revealed that Afro-American subjects had higher CRP levels compared to Caucasians (3.0 mg/L vs. 2.3 mg/L; *p* < 0.001), while women had higher levels than men (3.3 mg/L vs. 1.8 mg/L; *p* < 0.001). CRP levels > 3 mg/L were found in 31% of Caucasian men, 40% of Afro-American men, 51% of Caucasian women, and 58% of Afro-American women (*p* < 0.05 for each group vs. Caucasian men). Adjusting for conventional cardiovascular risk factors, estrogen use, statins, and body mass index, the prevalence of CRP levels > 3 mg/L remained significantly higher in Caucasian women (odds ratio [OR], 1.6; 95% confidence interval [CI], 1.1–2.5) and Afro-American women (OR, 1.7; 95% CI, 1.2–2.6), but not in Afro-American men (OR, 1.3; 95% CI, 0.8–1.9) compared to Caucasian men. This highlights the tendency of the populations different from Caucasian men (reference group) to exhibit elevated CRP levels [25]. 

The influence of the menstrual cycle stages on cardiovascular risk assessment has been investigated. Gaskins and colleagues conducted the BioCycle study in 2011, involving 318 women aged 18–44 years. In this study, they observed that CRP levels were the highest during the menstrual phase (0.74 mg/L), and the levels decreased during the follicular phase to reach their lowest point on the expected day of ovulation (0.45 mg/L) and increased again during the luteal phase. These variations were attributed to the relationship between CRP and hormonal changes, with a 10-fold increase in estradiol concentration associated with a 24% decrease in CRP levels. A 10-fold increase in progesterone levels during the luteal phase was associated with a 19% increase in CRP levels [26].

Recently, Evans and colleagues conducted a study to explore the association between CRP levels and stroke in 30,239 Afro-American and Caucasian individuals. During a follow-up period of 6.9 years, 292 ischemic strokes occurred among Afro-Americans and 439 among Caucasians. In Caucasians, an elevated risk was observed in the range of 3–10 mg/L, and even higher for CRP > 10 mg/L. However, among Afro-Americans, the association was only significant for CRP > 10 mg/L. The adjusted risk ratios for every standard deviation above the average CRP were 1.18 (95% CI, 1.09–1.28) overall, 1.14 (95% CI, 1.00–1.29) in Afro-Americans, and 1.22 (95% CI, 1.10–1.35) in Caucasians. This study further corroborates the differential predictive value of CRP in stroke risk assessment among individuals with different ethnic backgrounds, similar to that found in CAD [27].

## 5. Role of CRP in Predicting Cardiovascular Risk

When examining cardiovascular risk factors, the relationship between atherosclerosis and inflammation becomes evident. Sustained elevation of inflammatory markers is closely linked to the development of adverse cardiovascular events caused by the rupture of atherosclerotic plaques [28].

In 1998, Ridker and colleagues conducted a study to assess the predictive value of CRP measurement in combination with total cholesterol (TC) and high-density lipoprotein-cholesterol (HDL-C). In apparently healthy men who participated in the Physicians’ Health Study, the baseline levels of CRP, TC, and HDL-C were measured in 245 subjects who later experienced the first MI and 372 subjects who remained free of CVD during a 9-year follow-up period. Elevated levels of CRP, TC, and the TC/HDL-C ratio were significantly associated with a higher risk of future MI. Multivariate analysis showed that models incorporating CRP and lipid parameters were superior in predicting risk compared to models using only lipids. Furthermore, baseline CRP levels predicted the risk of MI even in individuals with low TC levels or high TC/HDL-C ratios [29]. Ridker and colleagues also studied CRP and LDL-C values to predict future cardiovascular events in 27,939 apparently healthy women during an 8-year follow-up period. Even though CRP and LDL-C showed a weak correlation (rho = 0.08), the baseline levels of each marker exhibited a strong linear relationship with the incidence of cardiovascular events. After adjusting for age, smoking, diabetes mellitus, blood pressure, and hormonal replacement therapy, ascending quintiles of CRP showed relative risks (RR) of 1.4, 1.6, 2.0, and 2.3 (*p* < 0.001) for a first cardiovascular event, compared to the lowest quintile. The corresponding RR for ascending quintiles of LDL-C were 0.9, 1.1, 1.3, and 1.5 (*p* < 0.001). CRP and LDL-C measurements identified different high-risk groups, demonstrating that the detection of both markers provided superior prognostic information than either marker separately [30].

Following the identification of the CRP value in predicting the first cardiovascular event in both men and women, its role in elderly populations remained to be studied. Cushman and colleagues measured baseline CRP levels in 3971 men and women aged >65 years without prior CVD. Approximately 26% of the participants had CRP concentrations >3 mg/L. After a 10-year follow-up, 547 participants developed CAD. The incidence of CAD was 33% in men and 17% in women with high CRP levels. Adjusted for age, ethnicity, and sex, the RR for CAD with CRP > 3 mg/L (compared to >1 mg/L) was 1.82 (95% CI, 1.46–2.28). After further adjustment for conventional risk factors, the RR decreased to 1.45 (95% CI, 1.14–1.86). Among men with a predicted risk of CAD between 10% and 20% according to the Framingham risk score, the observed incidence was 32% for individuals with high CRP. Among women with a predicted risk of CAD >20%, the observed incidence was 31% and 10% for high and normal CRP levels, respectively [31].

The predictive role of CRP in assessing stable ischemic cardiopathy has been investigated. Bogaty and colleagues conducted a study to measure CRP levels in 159 patients with established ischemic cardiopathy, utilizing 2 to 8 measurements taken at intervals spanning from 15 days to 6 years. The observed CRP values exhibited significant fluctuations across different ranges, namely, <1 mg/L, 1–3 mg/L, and >3 mg/L. The study revealed that 64 patients (40.3%) experienced a change in risk category between their initial and subsequent measurements. Variability in CRP levels remained consistent in different time points and clinical groups. Furthermore, this variability was found to be independent of conventional risk factors such as body mass index, smoking, and pharmacological treatment. These findings led the investigators to conclude that fluctuations in CRP levels among patients with stable ischemic cardiopathy may represent a limiting factor for accurate cardiovascular risk stratification [32].

In a longitudinal study encompassing 4257 patients, the utility of serial CRP measurements in risk stratification following an ACS was investigated. This study examined initial and subsequent increases in CRP levels over a 16-week period after an ACS. The results demonstrated that these changes were associated with a higher risk of major adverse cardiovascular events (MACE), cardiovascular death, and overall mortality, regardless of treatment. Therefore, serial CRP measurements following an MI can aid in identifying patients at increased risks of morbidity and mortality [33].

Zacho and colleagues conducted a comprehensive investigation on the potential causal association between CRP levels, ischemic cardiopathy, and stroke. The study included four distinct cohorts of individuals of white ethnicity with Danish ancestry. Researchers measured CRP levels and performed genotyping for four CRP polymorphisms. The findings of the study revealed that individuals with CRP levels exceeding 3 mg/L had a 1.6-fold increase in the risk of developing ischemic cardiopathy compared to those with CRP levels below 1 mg/L. Similarly, the risk of stroke was 1.3 times higher in individuals with CRP levels above 3 mg/L. A certain combination of CRP genotypes was associated with a significant 64% increase in CRP levels, although this elevation in CRP levels did not translate into a higher risk of ischemic vascular disease [34]. Another population-based study involving over 100,000 European individuals demonstrated a strong association between CRP gene polymorphisms and CRP levels. However, CRP locus variants were not associated with the incidence of CAD [35]. These findings suggest that although CRP genotypes can influence CRP levels, they may not directly contribute to the development of CVD.

## 6. Impact of CRP Levels on Cardiovascular Risk Assessment Algorithms

The incorporation of CRP into cardiovascular risk evaluation algorithms has gained significant attention due to its role in predicting major cardiovascular events. In particular, the Reynolds risk score was first developed to estimate the risk of adverse cardiovascular events over a 10-year period in women aged 45 and older. This scoring system considers factors such as age, systolic blood pressure, diabetes mellitus, and smoking, as well as levels of TC, HDL-C, and CRP. The Reynolds risk score helps identify individuals who may benefit from statin therapy to reduce their cardiovascular risk. Women with a Reynolds risk score of 10% or lower generally do not require statin therapy. Importantly, the Reynolds risk score reclassifies a substantial proportion (40–50%) of women who were previously classified as being at intermediate cardiovascular risk into either lower or higher risk categories, which differs from previous risk scoring models such as the Framingham risk score, which was primarily developed based on Caucasian male patients and does not incorporate CRP measurements [36].

Recognizing the independent association of CRP with future cardiovascular events and the improved predictive ability of risk calculators in women following its inclusion, Ridker and colleagues conducted a study involving 10,724 non-diabetic men, followed for an average time of 10.8 years. During this period, a total of 1294 cardiovascular events occurred. The study aimed to compare a traditional risk prediction model with one that incorporated CRP levels and family background (known as the Reynolds risk score for men). In comparison with the traditional model, the Reynolds risk score demonstrated better adjustment and successfully reclassified 17.8% of the study population into higher or lower risk categories, providing enhanced precision for the reclassified individuals. This study demonstrated, similar to previous findings in women, that the inclusion of CRP in a predictive model significantly improves the overall prediction of cardiovascular risk in men [37].

Coronary arterial calcium quantification has emerged as a novel approach to assessing cardiovascular risk. In 2018, a study involving 7382 individuals was conducted to validate a new risk scoring system including novel cardiovascular risk factors, such as CRP measurement and coronary calcium quantification. This model, known as the Astronaut Cardiovascular Health and Risk Modification (Astro-CHARM), demonstrated improved net reclassification of individuals with risks of developing significant cardiovascular events. Notably, the Astro-CHARM scoring system outperformed traditional risk factor equations, making it a potentially valuable tool for risk-based decision-making in CVD prevention [38].

In 2019, González-Pacheco and colleagues developed and internally validated a scoring system based on inflammation markers to predict mortality in 7396 Mexican patients with ACS. The scoring system incorporated leukocyte count, CRP levels, and serum albumin. Individually, each of these biomarkers was associated with an increased risk of in-hospital mortality. However, when combined into a composite score, there was a significant difference in hospital mortality rates across the four inflammation categories: no inflammation (1.8%), low (2.8%), medium (4.1%), and severe (13.8%) inflammation. After adjusting for multiple variables, the severe systemic inflammation category was found to be associated with a threefold higher risk of in-hospital mortality (OR, 3.02; *p* < 0.0001). In the entire cohort (the sum of derivation and validation sub cohorts), when stratifying patients based on the Global Registry of Acute Coronary Events (GRACE) risk score, the severe inflammation category was consistently associated with a significant increase in in-hospital mortality across all subgroups, particularly among patients with higher GRACE risk scores. The inflammation-based risk scoring system reclassified 25.3% of the population, resulting in a net reclassification index of 8.2% (*p* = 0.0001) [22].

These findings highlight the potential value of incorporating CRP measurement into cardiovascular risk evaluation models, offering improved risk stratification and reclassification. These advancements may contribute to our understanding of cardiovascular risk assessment and could have implications for enhancing clinical decision-making and preventive strategies.

## 7. Impact of Therapy on CRP Levels

Diverse therapeutic strategies, including dietary modifications and pharmacological interventions, have been associated with decreased serum CRP levels. It is possible that reducing systemic inflammation, as indicated by decreased CRP levels, may contribute to the reduction in CVD (Table 1).

### 7.1. Lipid Lowering Drugs

Several studies have demonstrated a significant reduction in serum and plasma CRP levels with the use of statins, independent of changes in lipid levels. The PRINCE study, comprising 1702 individuals in a primary prevention cohort, found that pravastatin significantly reduced the median CRP levels by 16.9% after 24 weeks compared to placebo. Multivariate analysis identified pravastatin and baseline CRP as the only predictors of the changes in CRP levels [39]. Another study evaluated the effects of three statins: simvastatin 20 mg/day, pravastatin 40 mg/day, and atorvastatin 10 mg/day on CRP levels. After 6 weeks of treatment, the CRP levels were significantly reduced with each statin. These reductions were similar between statins, and no relationship between CRP reduction and LDL-C levels was observed [40]. Similarly, the CARE study measured CRP levels initially and after 5 years in 472 participants randomly selected who remained free of recurrent coronary events during follow-up. This study found a strong correlation between CRP levels at baseline and those at 5 years (rho = 0.60; *p* < 0.001). Notably, patients assigned to placebo exhibited increasing CRP levels over time, whereas those receiving pravastatin experienced decreasing CRP levels, an effect persisting in multivariate analysis. The magnitude of CRP changes was not related to the changes in lipids among subjects treated with pravastatin [41].

The effect of atorvastatin on CRP levels and its implications for CVD in patients with type 2 diabetes was assessed by Soedamah-Muthu and colleagues. This study included 2322 patients and examined the CRP response after 1 year of atorvastatin treatment compared to placebo. The results revealed a significant net reduction of 32% in CRP levels among the group receiving atorvastatin, although there was considerable variability in individual CRP responses, with 45% of patients not experiencing a reduction in CRP levels. Interestingly, initial CRP levels did not predict the occurrence of CVD during the 3.8-year follow-up period, whereas initial LDL-C levels did. Consequently, this study does not support the use of CRP as an objective marker for statin therapy in patients with diabetes [42]. However, considering the association between elevated CRP levels and increased CAD, independent of hyperlipidemia, and the ability of statin therapy to reduce CRP levels regardless of its effect on lipid levels, it is plausible that statins may prevent ACS in individuals with elevated CRP levels. In a separate study involving 5472 participants without CAD over a 5-year period, lovastatin was found to reduce CRP levels by 14.8%, demonstrating efficacy in preventing coronary events in participants with reduced TC/HDL-C ratios but elevated CRP levels [43].

A randomized controlled trial including 4497 patients diagnosed with ACS was conducted to compare the effectiveness of early intensive (40 mg/day simvastatin for one month followed by 80 mg/day) and a delayed conservative (placebo for four months and then 20 mg/day of simvastatin) therapies using statins. After one month, patients in the early intensive therapy group experienced a significant reduction in LDL-C levels. However, there was no reduction in the primary outcomes, which included cardiovascular death, new ACS, or stroke. Interestingly, a clinical benefit was observed when serum CRP levels decreased, coinciding with the increase in simvastatin dosage from 40 mg/day to 80 mg/day [44]. In another clinical trial involving 4162 patients with ACS, the effectiveness of 80 mg/day of atorvastatin was compared to 40 mg/day of pravastatin. The study revealed a consistent decrease in CRP levels following the statin treatment, along with a reduced risk of recurrent MI or death caused by coronary factors. Notably, the correlation between CRP and LDL-C levels was found to be limited [45].

Regarding other lipid-lowering drugs, the addition of ezetimibe to simvastatin therapy resulted in an additional reduction of 16% (0.3 mg/L) in CRP levels [46]. This reduction in CRP levels was associated with a decrease in major cardiovascular events (RR, 0.73; 95% CI, 0.66–0.81). Collectively, these studies demonstrate that patients who achieved lower CRP levels after therapy experience improved cardiovascular outcomes, regardless of their TC or LDL-C levels. 

### 7.2. Glucagon-like Peptide-1 Receptor Agonists

Investigations focusing on the use of glucagon-like peptide-1 (GLP-1) receptor agonists, such as liraglutide and exenatide, have demonstrated significant improvements in cardiovascular risk factors among diabetic patients [47,48]. These GLP-1 receptor agonists exhibit a broad anti-inflammatory action, which is reflected in their ability to decrease CRP levels [49]. A controlled trial investigating the impact of liraglutide, in combination with metformin, on patients with CAD and recent diagnostic type 2 diabetes mellitus demonstrated a reduction in CRP levels. Notably, this effect was not observed with metformin monotherapy [50]. These findings were further supported by another study, which revealed that adding liraglutide was superior to lifestyle interventions in reducing CRP levels among patients with prediabetes or early diabetes who were treated with metformin [51]. Similarly, the addition of liraglutide to insulin treatment in obesity and diabetes patients resulted in significant reductions in CRP concentration after three months of treatment [49]. The effects of exenatide and semaglutide appear to be similar to that of liraglutide in terms of reducing CRP levels [49,52].

Dipeptidyl peptidase 4 inhibitors are a novel type of oral antidiabetic medications that have multiple effects, one of which is increasing the levels of circulating GLP-1. While these drugs have been shown to lower CRP levels, various well-controlled clinical trials have not been able to prove a decrease in the risk of adverse cardiovascular events [53].

### 7.3. Anti-Cytokine Agents

Recently, the impact of blocking inflammatory molecules using monoclonal antibodies on CVD has been explored. Canakinumab, an antibody that blocks IL-1β, was administered to 10,061 patients with ACS in the CANTOS study. The results revealed that compared to patients who received a placebo, those treated with canakinumab experienced a significant reduction in MACE such as MI, stroke, or cardiovascular death. Canakinumab had no effect on lipid levels, but it led to a decrease in CRP levels, with the extent of reduction depending on the dosage administered. In addition, patients who achieved a CRP level below 2 mg/L exhibited significant reductions in overall mortality, cardiovascular mortality, and MACE in comparison to patients who received placebo. Conversely, this reduction was not observed in patients whose CRP levels remained above 2 mg/L during treatment [54,55]. The CANTOS study was the first to demonstrate that an anti-inflammatory drug with no effects on lipid levels, can improve the progression and outcomes of patients with CAD and elevated CRP levels. Unfortunately, the high incidence of severe infections associated with canakinumab use has limited its application in CAD.

The inhibition of IL-6 may lead to significant decreases in CRP levels and improvements in cardiac and endothelial function parameters, although controlled clinical trials identifying its efficacy in primary and secondary prevention are still lacking [56,57,58]. Regarding TNF, extensive research indicates increased concentrations of this cytokine not only in the bloodstream but also within the myocardium of individuals with CVD, particularly heart failure [59]. However, attempts to mitigate TNF activity using etanercept or infliximab have not yielded favorable outcomes for patients. In fact, several clinical trials have clearly demonstrated an increased occurrence of detrimental cardiovascular events, particularly when TNF blockers are administered at high dosages [60,61]. Consequently, due to the evident cardiac toxicity associated with TNF inhibition, this therapeutic strategy is not used in the management of CAD.

### 7.4. Other Interventions

Several interventions used in the treatment of CAD or its primary risk factors, including thiazolidinediones [62], beta blockers [63], dietary regimens [64], and physical exercise [65], have been associated with a decrease in serum or plasma CRP levels. However, these interventions have not been evaluated in clinical trials to determine whether their cardiovascular benefits depend on the reduction of CRP. Neither acetylsalicylic acid nor colchicine reduces CRP levels [66,67].

## 8. Expanded Landscape of Inflammatory Markers in CAD

The fundamental involvement of inflammatory pathways in the initiation and progression of the atherogenic cascade has prompted a surge of interest in a broader spectrum of inflammation-related biomarkers within the context of CAD. Alongside the CRP, an array of easily accessible inflammatory markers has gained prominence. This repertoire encompasses conventional parameters such as leukocyte, neutrophil, and platelet counts, as well as neutrophil-to-lymphocyte and platelet-to-lymphocyte ratios. Additionally, other acute phase reactants, such as albumin, complement proteins, fibrinogen, and lipoproteins, have garnered attention as informative indicators of inflammatory status in CAD. The advent of automated reading platforms in clinical testing facilities has ushered in an era of discovery, unearthing a multitude of novel soluble inflammatory molecules that hold potential relevance for clinical cardiology. Noteworthy among these are proinflammatory cytokines including IL-6, IL-1β, TNF, IL-18, the monocyte colony-stimulating factor, and the platelet-derived growth factor. Alongside them, soluble molecules such as CD40 ligand, monocyte chemoattractant protein 1, osteoprotegerin, cystatin C, matrix metalloproteinases, and adiponectin have emerged as subjects of heightened interest [9,14,28,32,68].

While each of these molecules presents a compelling case as a prospective biomarker for CAD and some hold promise as viable therapeutic targets, it is imperative to acknowledge that none has yet attained the comprehensive support spanning basic, clinical, and translational investigations akin to that enjoyed by CRP. Hence, CRP continues to retain its status as the quintessential marker of systemic inflammation in the landscape of CAD.

## 9. Conclusions

The advent of highly sensitive assays for measuring CRP has provided us with a valuable biomarker that is cost-effective, easily accessible, and universally standardized, enabling widespread utilization in clinical practice. Over the past two decades, epidemiological studies have shed light on the significant role of CRP in CVD. Its predictive value, when combined with traditional risk factors, has prompted its inclusion in cardiovascular risk calculators. Given these attributes, CRP has become the preferred laboratory biomarker for personalized or precision cardiology, offering the potential to enhance risk assessment and refine therapeutic approaches tailored to individual patients.

## Figures and Tables

**Table 1:** Pharmacological interventions and their effects on the risk of CVD and CRP levels.

Drug	Primary Target	CVD Risk Reduction	CRP Reduction	Other Effects
Statins	3-hydroxy-3-methyl-glutaryl-CoA reductase	Yes	Yes	Hypolipemic
Ezetimibe	Nieman-Pick C1-like 1 (NPC1L1) protein	Yes	Yes	Hypolipemic, diminished insulin resistance
DPP-4 inhibitors	Dipeptidyl peptidase 4	No	Yes	Antidiabetic
GLP-1 agonists	Glucagon-like peptide-1 receptor	Yes	Yes	Antidiabetic, weight loss
Canakinumab	Interleukin-1β	Yes	Yes	Anti-inflammatory

## Data Availability

Not applicable.
